# Pharmacological Potential of the Standardized Methanolic Extract of *Prunus armeniaca* L. in the Haloperidol-Induced Parkinsonism Rat Model

**DOI:** 10.1155/2022/3697522

**Published:** 2022-09-29

**Authors:** Uzma Saleem, Liaqat Hussain, Faiza shahid, Fareeha Anwar, Zunera Chauhdary, Aimen Zafar

**Affiliations:** ^1^Department of Pharmacology, Faculty of Pharmaceutical Sciences, Government College University Faisalabad, Faisalabad, Pakistan; ^2^Riphah Institute of Pharmaceutical Sciences, Riphah International University, Raiwind Road Lahore, Pakistan; ^3^University Institute of Food Science & Technology, The University of Lahore, Raiwind Road Lahore, Lahore, Pakistan

## Abstract

Parkinson's disease (PD) is a complex, age-related neurodegenerative disease that causes neuronal loss and dysfunction over time. An imbalance of redox potential of oxidative stress in the cell causes neurodegenerative diseases and dysfunction of neurons. Plants are a rich source of bioactive substances that attenuate oxidative stress in a variety of neurological disorders. The aim of the present study was to evaluate the *Prunus armeniaca* L. methanolic extract (PAME) for anti-Parkinson activity in rats. PD was induced with haloperidol (1 mg/kg, *IP*). The PAME was administered orally at 100, 300, and 800 mg/kg dose levels for 21 days. Behavioral studies (catalepsy test, hang test, open-field test, narrow beam walk, and hole-board test), oxidative stress biomarkers (SOD, CAT, GSH, and MDA) levels, neurotransmitters (dopamine, serotonin, and noradrenaline) levels, and acetylcholinesterase activity were quantified in the brain homogenate. Liver function tests (LFTs), renal function tests (RFTs), complete blood count (CBC), and lipid profiles were measured in the blood/serum samples to note the side effects of PAME at the selected doses. Histopathological analysis was performed on the brain (anti-PD study), liver, heart, and kidney (to check the toxicity of PAME on these vital organs). Motor functions were improved in the behavioral studies. Dopamine, serotonin, and noradrenaline levels were significantly increased (*P* < 0.001), whereas the level of acetylcholinesterase was decreased significantly (*P* < 0.001). The levels of superoxide dismutase (SOD), catalase (CAT), and reduced glutathione (GSH) were increased, while malondialdehyde (MDA) and nitrite levels were decreased in the PAME-treated groups significantly compared with the disease control group, hence reducing oxidative stress. The incidence of toxicity was determined by biochemical analysis of LFT and RFT biomarkers testing. The histopathological analysis indicated that neurofibrillary tangles and plaques decreased in a dose-dependent manner in the PAME-treated groups. Based on the data, it is concluded that PAME possessed good anti-Parkinson activity, rationalizing the plant's traditional use as a neuroprotective agent.

## 1. Introduction

Neurological diseases are a divergent group of diseases of the sensory system that include the brain, peripheral nerves, and spinal cord [1]. Proteostasis, strain, neuroinflammation, apoptosis, and oxidative stress are involved in the pathology of neurodegenerative disorders, which are all responsible for continuous neuronal damage and destruction [2]. Dementia, Parkinson's disease (PD), and motor neuron dysfunction are important neurodegenerative disorders. It is estimated that, in Pakistan, 450,000 people are suffering from PD. According to the WHO, the death rate associated with PD in Pakistanis is 1.87% of the total population.

PD is a complex neurodegenerative disease that happens due to continuous damage of dopaminergic neurons projecting from the substantia nigra (pars compacta) to the corpus striatum. The disease was explicated for the first time by Dr. James Parkinson in 1817 in his “Essay on the shaking palsy”. There are two forms of PD: familial (genetically inherited) and sporadic (idiopathic). It is a slowly progressive degenerative disorder with both motor and nonmotor features. Signs and symptoms of PD include tremor at rest, postural or gait abnormalities, muscle rigidity, and bradykinesia [3]. The pathological characteristic of PD is Lewy bodies. They are *α*-synuclein-immunoreactive clusters of proteins that majorly cause proteolysis. These include ubiquitin and reduction in the level of dopaminergic neurons in the striatum, expressed as a reduction in voluntary movements. As PD progresses, the spread of Lewy bodies expands to the neocortical and cortical regions [4].

The disease usually begins at the age of 65 to 70 years and is more occurring in men than women [5]. The neuropathological mechanisms of PD are multifactorial and involve genetic as well as nongenetic and environmental factors. Protein aggregate accumulation, mitochondrial damage, impaired protein clearance pathways, neuroinflammation, oxidative stress, excitotoxicity, and genetic mutations are the main pathological mechanisms [6]. Cases of PD involve genetics-based origin, only constituting 5–10 in number out of 100 cases. Some of the variant genes control a cluster of molecular pathways, and when they are disturbed, they cause neurological dysfunction leading to PD [7]. Tremendous genome-wide association studies (GWASs) propose a specific acquired encoding for proteins that are linked to these molecular pathways and play a role in sporadic PD. Examples of different pathways are mitochondrial function abnormality and neuronal inflammation [8].

The current medication of PD treats only symptoms; neither slows down the progression of the disease nor halts the dopaminergic neuron degradation [9]. Regarding the treatment of PD, many guidelines are available in which dopamine agonists are used in treating young-onset patients and levodopa for older patients. For initial therapy, patients who go through first motor fluctuations, MAO-B inhibitors act as a better treatment option. Similarly, COMT inhibitors enhance the action of levodopa if any wearing-off symptoms present [10]. Treatments such as pharmacological dopamine replacement and the use of deep brain stimulation are extremely effective. In recent decades, PD has been effectively managed by enhancing one's quality of life [11]. Different strategies will be developed in the future to identify people who are at high risk to develop PD [12]. Newer formulations are also under development to enhance the efficacy and reduce the toxicity of available drugs [13]. A novel compound such *β*-asarone also showed good potential against PD [14].

There are a number of plant species that have been recognized to have excellent therapeutic potential against neurodegenerative disorders [4]. These have been found to exert a diverse range of protective effects that mitigate the devastating neurodegeneration [15]. Generally, plant species with antioxidant properties have been broadly recognized to ameliorate the disease process [16–19]. *P*. armeniaca L. contains various flavonoids such as quercetin, a potent antioxidant that has been credited to protect neurons from free radical-associated cellular degeneration in PD [20]. However, there is a long list of isolated bioactive compounds from therapeutic plants that have been found to ameliorate the neurodegenerative disorders [21].

*P. armeniaca* L. (apricot) is a significant plant species from the family *Rosaceae* and is traditionally used for treating different ailments [22]. This plant is found in China, Turkey, Iran, Italy, France, Morocco, Pakistan, Spain, USA, and India [23]. Apricots are high in oils, proteins, fatty acids, vitamins, carotenoids (*β*-carotene, *γ*-carotene), minerals (calcium, Na^+^, K^+^, PO_4_^3-^, Mg^2+^, Fe^2+^, Zn^2+^), polyphenols, and flavonoids [24,25]. According to the Unani system, it is utilized as an antidiarrheal, emetic, and anthelmintic in liver disorders, ear infection, and deafness and as an expectorant for dry throat, laryngitis, lung ailments, and abscesses [24]. The present study was designed to assess the potential of *P. armeniaca* L. methanolic extract (PAME) for the management of PD on the basis of scientific grounds by using a haloperidol-induced PD animal model.

## 2. Materials and Methods

### 2.1. Plant Collection and Identification

Fresh kernels of *P. armeniaca* were obtained from the local market of Faisalabad, they were verified by the Botany Department of Government College University Faisalabad by Taxonomist Dr. Qasim Ali under authentication number 279-BOT-21, and the voucher specimen was placed in the herbarium.

### 2.2. Plant Extract Preparation

The fresh kernels were collected, washed to remove filth, superfluous substances, and dried under shade for 1 month. Then, the dried kernels were crushed into powder by utilizing an electronic blender. The powder of *P. armeniaca* was weighed in three beakers of 1000 mL capacity. The microwave was adjusted at 9000 watts. In the 1st cycle, 100 g of powder and 750 mL methanol were added in all beakers. Then, these beakers were placed in a microwave oven and the oven was heated for two minutes; then stopped the oven and opened it for 30 seconds. This specific method was repeated for 5 times. At the end, the supernatant was filtered by using filtered paper or muslin cloth. In the 2nd cycle, again 500 mL methanol was added in each beaker and then all beakers were placed in a microwave oven. The oven heated up for 2 minutes and stopped for 30 seconds. The same method was repeated for 5 times. And the supernatant was filtered by using muslin cloth. In the 3rd cycle, 500 mL methanol was added in each beaker, and the same method was repeated for 5 times. Filtrates were pooled in the same reservoir. In the rotary evaporator, the distillate was evaporated under reduced pressure at 4 rpm. After the evaporation of the solvent, the semisolid material of *Prunus armeniaca* L. methanolic extract (PAME) was formed [4].

### 2.3. Plant Characterization

#### 2.3.1. Physicochemical Analysis

The physical and chemical properties of the powder plant material were investigated to calculate the moisture content, total ash, acid indissoluble ash, aqueous indissoluble ash, water, alcohol dissoluble extractives, and sulfated ash content; all these analysis were performed according to established protocols and already performed in our previous published articles [26].

#### 2.3.2. Phytochemical Analysis

To investigate the alkaloid, phenolic, and flavonoid contents of the plant extract, the phytochemical analysis was performed according to previous published protocols [4,26].

*Estimation of Total Phenolic Content (TPC).* Folin–Ciocalteu's phenol reagent (0.2 mL) was mixed in the test tubes with the sample and standard solution (0.2 mL), and after 4 minutes, 1 mL of sodium bicarbonate solution (15%) was added into these test tubes. For the next 2 hours, the solution was kept at room temperature, and various concentrations of standard reference at 10, 20, 30, 40, 50, and 60 *μ*g/mL were formed to build up a linear regression equation. Then, the absorbance was taken at 760 nm. The test tube standard had all reagents except the analyte, and for drawing the standard curve gallic acid was used [27]. As a result, the total phenolic content of the test sample was measured as mg of gallic acid equivalents (GAEqs)/g of the extract and is calculated using the following equation:

(1) $$\begin{array}{c} \text{total phenolic content}=\frac{\text{gallic acid equivalents}\times \text{extract volume}}{\text{sample }\left(g\right)}. \end{array}$$

*Estimation of Total Flavonoid Content (TFC).* 10% aluminum nitrate solution (0.1 mL), 1 M potassium acetate (0.1 mL), and 4.6 mL distilled water were mixed with the sample (0.2 mL) and standard solution (0.2 mL) in test tubes (0.2 mL). Then, for the next 45 minutes, the mixture prepared was placed at room temperature for incubation. The test tube standard has all reagents except analyte, and quercetin (QTN) will be used for drawing the standard curve. Standard curves for reference sample solution were taken at different concentrations 10, 20, 30, 40 50, and 60 *μ*g/mL and at 415 nm, and the absorbance was measured [27]. The TFC was measured as mg of quercetin equivalents/g (QEqs/g) of the extract and is determined using the following equation:

(2) $$\begin{array}{c} \text{total flavonoid content}=\frac{\text{QTN equivalents}\times \text{extract volume}}{\text{sample }\left(g\right)}. \end{array}$$

*Estimation of Total Alkaloids.* The gallic acid solution in 5% methanol (100 L), concentrated H_2_SO_4_ (2 L), and sample/standard alcoholic solution (100 L) were combined in a test tube. For the next 10 minutes, the solution was boiled, and at 660 nm the absorbance was measured. The test tube standard has all reagents except the sample. For drawing the standard curve, atropine was used. Standard atropine solutions at different concentrations 10, 20, 30, 40, 50, and 60 *μ*g/mL were made. Presence of total alkaloids in the extract was measured as milligrams of atropine equivalents (AEqs/g) of the extract, and total alkaloids are figured out using the following equation [28]:

(3) $$\begin{array}{c} \text{total alkaloids}=\frac{\text{piperine equivalents}\times \text{extract volume}}{\text{sample }\left(g\right)}. \end{array}$$

*Determination of Antioxidant Activity Using DPPH Assay.* Various concentrations of the sample will be used to determine the antioxidant activity. DPPH will be dissolved in methanol. In a total volume of 4 mL methanol, 200 *μ*L of 0.05% DPPH will be mixed with 80 *μ*L of the sample. And for the next 30 minutes it was kept in darkness. The results will be expressed as a percentage of inhibition using the percent inhibition relationship [22]:

(4) $$\begin{array}{c} \text{\%scavenging effect}=\frac{\text{absorbance of control}-\text{absorbance of sample}}{\text{absorbance of control}}\times 100\text{.} \end{array}$$

### 2.4. Experimental Animals

Thirty-six healthy albino Wistar rats weighing 150–200 g of either sex were used for *in vivo* anti-Parkinson activity. The rats were acquired and placed in an animal house under proper conditions (12-hour light and dark cycle, room temperature 25°C) in polypropylene cages. Animals were divided into 6 groups after one-week normal feeding with water ad libitum. The study was started after getting ethical approval (Ref. No. GCUF/ERC/2222) from the Institutional Review Board of Government College University Faisalabad ruling under the regulation of the Institute of Laboratory Animal Resources, Commission on Life Sciences, National Research Council (1996).

### 2.5. Evaluation of Anti-Parkinson Activity

#### 2.5.1. Disease Induction

In rats, haloperidol (1 mg/kg) was given once daily (intraperitoneally) for twenty-one days to induce Parkinson's disease except the normal control group. It was injected before one hour of extract treatment [15].

#### 2.5.2. Study Design

Thirty-six healthy albino Wistar rats (almost equal from each sex) were divided into 6 groups (*n* = 6 each group). Group 1 was served as normal control and was given the vehicle only. Group 2 or the disease control group received 1 mg/kg of haloperidol via intraperitoneal injection. Group 3 or the standard group received 100 mg/kg of levodopa and 25 mg/kg carbidopa [29]. Groups 4, 5, and 6 or treatment groups received the kernel extract of PAME at doses 100, 300, and 800 mg/kg.

All the groups were treated for consecutive 21 days. Behavioral and weight alterations were measured at the beginning, during, and end of the study. After 21 days of treatment, all the rats were euthanized and observations were recorded by biochemical and histological experiments. Brains from all the groups were separated and rinsed with normal saline and preserved in chilled phosphate buffer (pH 7.4) for measurement of neurotransmitters (dopamine, serotonin, norepinephrine) and oxidative stress biomarkers. Blood samples of the animals were taken by cardiac puncture. LFTs and RFTs were performed on blood plasma and serum, respectively. The vital organs such as brain, liver, heart, and kidney were preserved in 10% buffered formalin for further toxicity studies and histopathological observations.

#### 2.5.3. Behavioral Analysis

The catalepsy, open field, hang, hole board, swim, narrow beam walk, elevated plus maze, and Y-maze tests were conducted to investigate the behavioral analysis [29].

*Catalepsy Test*. Catalepsy is the inability of rats to response to external stimuli and rigidity of muscles. Briefly, the rats were placed on a wooden bar (1 cm diameter and 3–9 cm elevated) with their forelimbs after giving haloperidol and the time taken to correct the imposed posture was recorded as a catalepsy indicator. Catalepsy ends when either the rats climb up the bar or their forelimbs touched the floor. These observations were noted after 30, 60, 90, and 120 minutes [29]. All findings were done in a quiet environment at 23–25 degrees Celsius, with a 5-minute cut-off time [30].

The scoring of catalepsy was as follows:

- Score 0: when the rats were placed on the table, they moved normally
- Score 0.5: when pushed or touched, the rats behaved normally
- Score 2: in 10 seconds, the rats were unable to correct the imposed posture; 1 score for every paw

*Open-Field Test*. This test was conducted to evaluate the locomotor and exploratory behavior patterns of experimental rats. For this test, a square-shaped wooden box (100 cm width × 100 cm diameter × 45 cm height) was made of plywood material painted with white color and black-line-coated floor has been splinted into 25 blocks. Ethanol was used to wash the apparatus. For 5 minutes, the rats were positioned in the center of the field and let them enter the box freely, and then both central and horizontal numbers of squares crossed and the number of crossings, time of stretch attend, defecation, freezing, and postures were recorded [29].

*Hole Board Test*. For the evaluation of behavioral components of the experimental animals, the hole board test is used. The apparatus consisted of a (30 cm length (L) × 30 cm width (W)) square-shaped area that contained 16 equidistant holes. On the 20th day of dosing, each animal was acclimatized for 30 minutes near the apparatus before being placed in the center of the hole board apparatus for the evaluation of test. Focused (edge sniffing and head dipping), horizontal (walking and immobile sniffing), and vertical (climbing and rearing) exploratory activities, as well as immobility events, were observed during a 120-second session with each animal [4].

*Narrow Beam Walk Test*. This said test was carried out to analyze the motor coordination and balance in the Parkinson's model. The rats were taught to walk for 120 seconds through a narrow stationary flat wooden plank measuring 100 cm in length and 4 cm in width. The time it took to cross a narrow beam with two ends pointing in opposite directions was recorded as a measure of motor coordination and balance [29].

*Swim Test*. It is the characteristic test for the observation of depression in the animal, therefore called behavior despair test. The test was designed in a cylindrical setting (16 cm H × 40 cm L × 25 cm W) that was filled with water to check the immobility or mobility potential of the animal. The typical observation of this test was that the animal remains immobile with its head above the surface of water. It was a 2-day procedure, after the first trial on 20th day, and the final trial was done on the 21st day of experiment. The rats were placed individually in a cylinder containing 19 cm of water, and the temperature was kept constant at 23°C. Each animal was subjected to a 6-minute swim test. The swimming behavior was examined, and the duration of immobility in the last four minutes was recorded, followed by 2 minutes of acclimatization [31].

*Y-Maze Test*. In the Y-maze test, spontaneous alterations were used as an index of working short-term memory. The maze was considered of a triangular central area with three A, B, and C equilateral arms of (35 cm L × 25 cm H × 10 cm W) each rat was placed in the central area facing one of the arms. When all four paws of the rat were in the arm, the entry was scored. Consecutive entries (ABC, BCA, or CAB but not CAC) into all three arms were defined as spontaneous alterations in behavior. Maximum spontaneous alteration (total number of arms entered) and percentage of spontaneous alteration (actual/maximum alterations × 100) were calculated [32].

#### 2.5.4. Evaluation of Biochemical Parameters

The levels of oxidative stress biomarkers GSH, SOD, CAT, and MDA were quantified in the brain, liver, heart, and kidney homogenates; whereas acetylcholinesterase activity and dopamine, serotonin, and norepinephrine levels were measured in the brain tissue homogenate [15].

Established protocols as already published and employed in our previous studies were followed for the estimation of catalase activity (CAT), dismutase activity (SOD), glutathione (GSH), and malondialdehyde (MDA) levels in brain homogenates [29].

#### 2.5.5. Estimation of Total Protein

**Solution A** was prepared by adding 2% Na_2_CO_3_ in 0.1 N NaOH; **solution B**, 1% sodium potassium tartrate in H_2_O; **solution C**, 0.5% CuSO_4_ in H_2_O; **reagent 1**, 48 mL of solution A, 1 mL of solution B, and 1 mL of solution C; and **reagent 2**, 1 part of Folin phenol in part of water in 1 : 1 ratio.

Bovine serum albumin (BSA) 0.2 mL was added as a standard in 5 test tubes, and 0.8 mL distilled water was added. Then, in each test tube, reagent 1 (4.5 mL) was added and incubated for 10 minutes. Following the initial incubation, 0.5 mL of reagent 2 was added and incubated for another 30 minutes. At 660 nm, the absorbance was taken and a standard graph was drawn. The standard curve was constructed by BSA, and the values were expressed in mg/mL [33]. The following regression line was used:

(5) $$\begin{array}{c} \text{absorbance }\left(Y\right)=0.00007571x+0.00004762. \end{array}$$

#### 2.5.6. Measurement of Nitrite Levels

Quantification of nitric oxide can be done in the form of nitrite levels. The tissue homogenate and Griess reagent (36 mL) were mixed in equal amounts and incubated for 10 minutes for this assay. The absorbance of the reaction mixture was measured at 546 nm [4,29]. The following regression line was used:

(6) $$\begin{array}{c} \text{absorbance }\left(Y\right)=0.003432+0.0366.\; \end{array}$$

#### 2.5.7. Evaluation of Acetylcholinesterase Activity

In this assay, 2.6 mL phosphate buffer (pH 8) was added to (0.1 M) 2,4-di-thio-bis-nitrobenzoic acid (100 L) and 0.4 mL tissue homogenate was mixed with acetylthiocholine iodide (20 L). The resultant mixture became yellow in appearance due to the reaction of DTNB with acetylthiocholine iodide. At 412 nm, the absorbance was recorded. Change in absorbance was measured after every 2 minutes till the 10 minutes of duration [4,34]. AChE activity was determined by using the following formula:

(7) $$\begin{array}{c} R=5.74\times 10^{-4}\;\times \frac{A}{C^{{}^{\circ}}}, \end{array}$$

where C_O_ is the original concentration of tissue (mg/mL), A is expressed as the variation in absorbance/minute, and R is the rate in moles of substrate hydrolyzed/min/gram of tissue.

#### 2.5.8. Tests for the Estimation of Neurotransmitter Levels

*Aqueous Phase Preparation*. The brains of all sacrificed rats were isolated and weighed. The homogenate of brain tissue was preserved in 5 mL of HCL-butanol and centrifuged for 10 minutes at 2000 rpm. After centrifugation, an aliquot of the supernatant was taken, 0.31 mL of HCL (0.1 M) and 2.5 mL of heptane were added, and the mixture was shaken for 10 minutes. The mixture was centrifuged at 2000 rpm for 10 minutes. Two layers were separated after centrifugation, and the temperature was kept at 0°C throughout the procedure. The liquid phase (0.2 mL) was used for the determination of neurotransmitter levels [35].

*Estimation of Serotonin Levels*. 0.25 mL O-phthalaldehyde was added to the liquid phase (0.2 mL). To make a fluorophore, the prepared solution was heated to 100°C. The solution was then allowed to cool to room temperature before the absorbance was measured. The emission wavelength for the fluorescence method is 340 nm, and the excitation wavelength is 305 nm [34].

(8) $$\begin{array}{c} \text{Absorbance}\left(Y\right)=0.0299x+0.0918. \end{array}$$

*Estimation of Dopamine and Noradrenaline Levels.* A 0.2 mL sample of the aqueous phase was mixed with 0.05 mL HCL (0.4 M) and 0.1 mL sodium acetate/ethylenediaminetetraacetic acid buffer (pH 6.9). After mixing, 0.1 mL of Na_2_SO_3_ solution was added to carry out the oxidation reaction for 1.5 minutes, followed by 0.1 mL of acetic acid. The solution was heated to 100°C for 6 minutes before cooling to room temperature. The absorbance of dopamine and noradrenaline was measured at 350 nm and 450 nm, respectively [34].

(9) $$\begin{array}{c} \text{absorbance of dopamine }\left(Y\right)=0.0314x+0.1067. \end{array}$$

### 2.6. Histopathological Analysis

The brains of all animals were silver stained, while other organs such as liver, heart, and kidney were also processed for by using H&E staining [36].

### 2.7. Statistical Analysis

The results were presented as means ± SEM. In GraphPad Prism, version 6, data were analyzed using one-way/two-way ANOVA followed by Bonferroni's multiple comparison as a post hoc test, with *P* values of <0.05 considered significant.

## 3. Results

### 3.1. Plant Characterization

#### 3.1.1. Physicochemical Analysis

PAME was characterized for physicochemical parameters that are shown in Table 1. The moisture content was found to be 7%, the total ash content was 22%, and the sulfated ash content was 52%. The water-insoluble ash content (39%) was found higher compared with alcohol-insoluble ash (19%); while water- and alcohol-soluble extractives were 1.6% and 5.8%, respectively.

#### 3.1.2. Phytochemical Analysis

Phytochemical screening of plant extracts showed different quantities such as polyphenolic content (26.07 ± 0.71 mg/g), total alkaloid content (43 ± 0.28 mg/g), and total flavonoids (70.66 ± 0.45 mg/g). These constituents may be possibly responsible for the biological activities of *P. armeniaca* L. Alkaloids were quantified using the gallic acid standard curve with linear regression equation “*y* = 0.0006*x* and *R*^2^ = 0.9957,” while flavonoids and polyphenols were quantified using the quercetin standard curve with linear regression equation “*y* = 0.001*x* and *R*^2^ = 0.9748” and piperine standard curve with linear regression equation “*y* = 0.00396*x* and *R*^2^ = 0.9954,” respectively. The results are expressed in Table 2.

#### 3.1.3. Determination of Antioxidant Activity Using DPPH Assay

The antioxidant activity of the phytoconstituents analyzed increased as the concentration of the extracts increased, according to the 2,2-diphenyl-1-picrylhydrazine (DPPH) finding. From the curve drawn between concentration and residual DPPH, the extract's IC_50_ value was determined. The plant extract had an IC_50_ value of 60.32 ± 2.73, while ascorbic acid had an IC_50_ value of 47.51 ± 1.92.

### 3.2. Behavioral Analysis for the Evaluation of Anti-Parkinson's Effect of PAME

#### 3.2.1. Catalepsy Test

**Figure 1:**
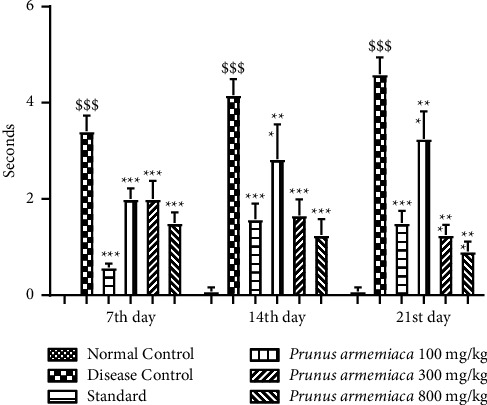
Effects of *P. armeniaca* L. on measurement of cataleptic scores. Data are presented as mean ± SEM (*n* = 6), where ^$^*P* < 0.05, ^$$^*P* < 0.01, and ^$$$^*P* < 0.001 compared with the normal control group. ^*∗*^*P* < 0.05, ^*∗∗*^*P* < 0.01, and ^*∗∗∗*^*P* < 0.001 compared with the disease control group.

On the 7th, 14th, and 21st days, all experimental rats in the respective groups were tested for cataleptic reaction. Each animal's cataleptic reaction was recorded at 30, 60, 90, and 120 minutes following treatment in all experimental groups. The results are expressed in Figure 1. In contrast to the normal control group, the time spent substantially (*P* < 0.001) rose in the haloperidol (HAL) group, which was noted on the 7th, 14th, and 21st days of treatment. In comparison with the group treated with haloperidol only, the induced cataleptic outcome of haloperidol was dose- and time-dependent (*P* < 0.001) reversed by oral administration of levodopa and carbidopa (standard anti-parkinsonian drugs). The cataleptic response was reduced in all PAME-treated groups in a dose-dependent manner. When compared with the disease group, the 800 mg/kg dose demonstrated a significant (*P* < 0.001) reduction in catalepsy and the lowest score was noted on the 21st day.

#### 3.2.2. Narrow Beam Walk Test

**Figure 2:**
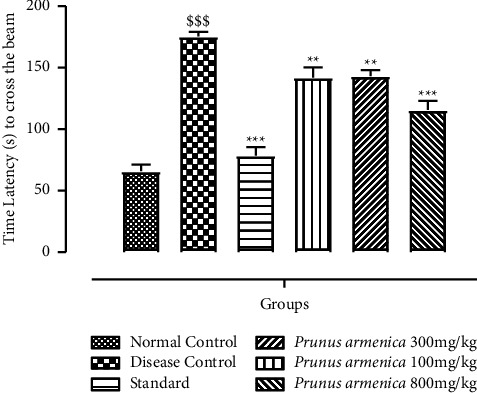
Effects of *P. armeniaca* L. on time latency (seconds) in the narrow beam walk test. Data are presented as mean ± SEM (*n* = 6). ^$$$^*P* < 0.001 compared with the normal control group. ^*∗∗*^*P* < 0.01 and ^*∗∗∗*^*P* < 0.001 compared with the disease control group.

The latency period for a narrow beam test conducted was examined as a marker of motor function as well as coordination on the 21st day following the administration of doses, whereas the number of padded faults was utilized to measure balance. The delay time and number of foot mistakes in the haloperidol-treated group were substantially (*P* < 0.001) higher than those in the normal control group. Therapy with levodopa and carbidopa reduced the crossing time approximately identically to the control group (*P* < 0.001), indicating that treatment eliminated the haloperidol effect. Compared with the disease control group, the 100 and 300 mg/kg extract-treated groups had substantially shorter latency times and fewer foot slip mistakes (*P* < 0.01). When compared with the disease control group, the 800 mg/kg dosage level of PAME significantly (*P* < 0.001) reduced the number of foot slip mistakes. The results are shown in Figure 2.

#### 3.2.3. Open-Field Test

**Figure 3:**
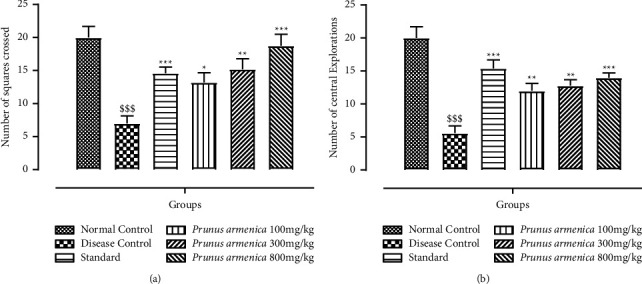
Effects of *P. armeniaca* L. on the (a) number of squares crossed and (b) number of central explorations in the open-field test. Data are presented as mean ± SEM (*n* = 6). ^$$$^*P* < 0.001 compared with the normal control group. ^*∗*^*P* < 0.05, ^*∗∗*^*P* < 0.01, and ^*∗∗∗*^*P* < 0.001 compared with the disease control group.

The effects of PAME on the number of lines crossed and exploratory behavior are shown in Figures 3(a) and 3(b). The treatment groups had a significant influence on the number of squares traversed, as well as the central and peripheral explorations (*P* < 0.001). Post-test analysis revealed that plant extract at 100 mg/kg and 300 mg/kg resulted in a substantial increase (*P* < 0.01) and the most significant improvement (*P* < 0.01) in central area explorations. Although, in terms of the number of squares traversed, animals in the plant-treated groups showed dose-dependent recovery, *P* < 0.05, *P* < 0.01, and *P* < 0.001, respectively, at all treatment dosages. As a result, the rats demonstrated a dose-dependent increase in locomotor activity, as well as an increase in exploratory behavior and crossings. An indication of anxiolytic impact was a substantial (*P* < 0.001) reduction in the frequency of both central and horizontal explorations in the disease control group.

#### 3.2.4. Hole Board Test

**Figure 4:**
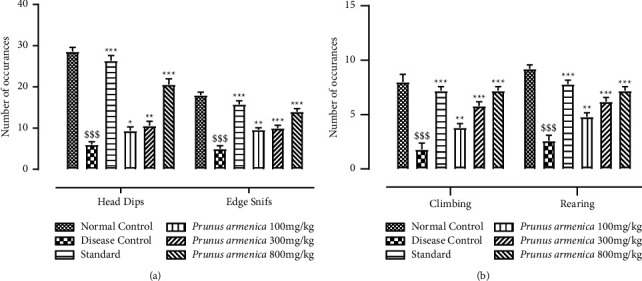
Effects of *P. armeniaca* L. on the (a) number of focused exploratory activities and (b) number of vertical exploratory activities in the hole board test. Data are presented as mean ± SEM (*n* = 6). ^$$$^*P* < 0.001 compared with the normal control group. ^*∗*^*P* < 0.05, ^*∗∗*^*P* < 0.01, and ^*∗∗∗*^*P* < 0.01 compared with the disease control group.

The hole board test was performed to evaluate the antianxiety potential and focused horizontal and vertical exploratory activities in all the groups of experimental animals. And the number of rats putting their heads into the holes is a sign of curiosity. Furthermore, compared with the only haloperidol-treated group, post hoc analysis indicated a substantial (*P* < 0.001) reduction in the number of hole exploration. The number of focused (head dipping, edge sniffing) and vertical exploring behaviors decreased significantly (*P* < 0.001) in the disease group (Figures 4(a) and 4(b)). All the treatment groups showed an increase in their exploratory behaviors, with the 100 mg/kg and 300 mg/kg dosage groups showing fairly significant recovery (*P* < 0.01) and the 800 mg/kg dose group of PAME showing a substantial *P* < 0.001) improvement in climbing and rearing. The plant extract's dose-dependent impact suggests that it can protect against dopaminergic depletion.

#### 3.2.5. Swim Test

In the Parkinson disease model, the severity of parkinsonian signs was evaluated through swimming scores after haloperidol and treatment dose administration as displayed in Table 3. All the treatment groups increased their exploratory behaviors, with the 100 mg/kg and 300 mg/kg dosage groups showing fairly significant recovery (*P* < 0.01) and the 800 mg/kg dose group of PAME showing a substantial (*P* < 0.001) improvement in climbing and rearing. The plant extract's dose-dependent impact suggests that it can guard against dopaminergic depletion. In addition, a dose-related significant reduction (*P* < 0.001) in the duration of immobility was noticed, compared with the disease control group. The standard treatment group also showed a significant (*P* < 0.001) improvement in swimming. In addition, compared with the disease control group, there was a dose-related substantial decrease (*P* < 0.001) in the length of immobility. Swimming performance was also significantly improved (*P* < 0.001) in the standard treatment group.

#### 3.2.6. Y-Maze Test

**Figure 5:**
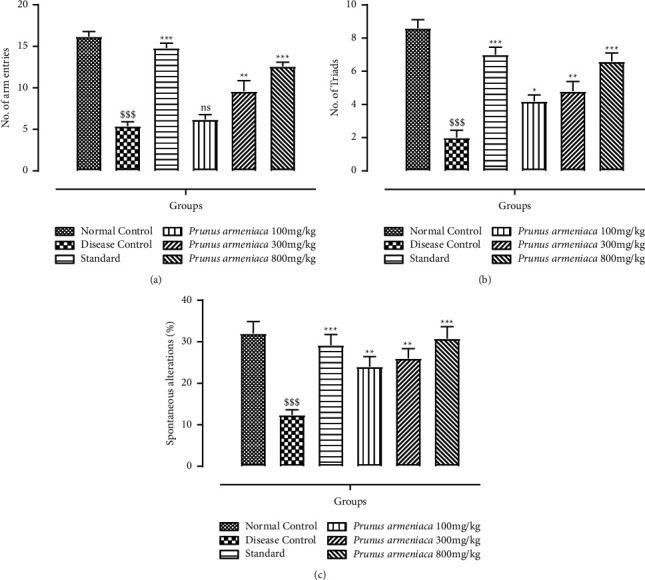
Effects of *P. armeniaca* L. on (a) arm entries, (b) the number of Triads, and (c) spontaneous alterations (%) in the Y-maze test. Data are presented as mean ± SEM (*n* = 6). ^$$$^*P* < 0.001 compared with the normal control group. ^*∗*^*P* < 0.05, ^*∗∗*^*P* < 0.01, and ^*∗∗∗*^*P* < 0.001 compared with the disease control group.

The impact of PAME on short-term memory or working memory was evaluated using the spontaneous alternation behavior Y-maze test, as stated previously. In comparison with the normal control group, a significant (*P* < 0.001) decrease in spontaneous alternation behavior after haloperidol administration was revealed. The decrease in the relative proportion of spontaneous alternation behavior caused by haloperidol was significantly reversed dose dependently (*P* < 0.05, *P* < 0.01, and *P* < 0.001) upon administration of PAME (100, 300, and 800 mg/kg). Haloperidol administration also reduced the number of arm entries in the disease control group. The treatment groups, on the other hand, showed a significant (*P* < 0.001) recovery of memory loss. The percentage of spontaneous alterations increased significantly (*P* < 0.001) in the standard and 800 mg/kg dosage groups. All the results are shown in Figures 5(a)–5(c).

### 3.3. Estimation of Neurotransmitter Levels in the Brain

#### 3.3.1. Estimation of Dopamine and Noradrenaline Levels

**Figure 6:**
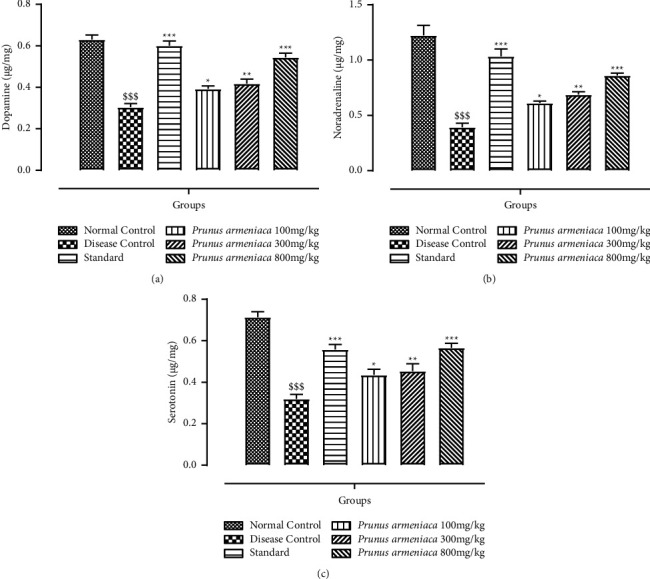
Effects of *P. armeniaca* L. on (a) dopamine, (b) noradrenaline, and (c) serotonin levels in brain homogenates. Data are presented as mean ± S.E.M. (*n* = 6). ^$$$^*P* < 0.001 compared with the normal control group. ^*∗*^*P* < 0.05, ^*∗∗*^*P* < 0.01, and ^*∗∗∗*^*P* < 0.001 compared with the disease control group.

The levels of dopamine and noradrenaline were measured in brain's tissue homogenate. The treatment groups, on the other hand, showed a significant (*P* < 0.001) recovery of memory loss. The percentage of spontaneous alterations increased significantly (*P* < 0.001) in the standard and 800 mg/kg dosage groups. In the standard as well as plant extract-treated groups, a significant improvement was seen in the level of both neurotransmitters, which was a comparable potential to the haloperidol-treated group. However, all the treatment groups exhibited a dose-dependent recovery in the levels of dopamine and noradrenaline (*P* < 0.05, *P* < 0.01) at 100 mg/kg and 300 mg/kg, with a significant (*P* < 0.001) increase at the highest dose level 800 mg/kg, as shown in Figures 6(a) and 6(b).

#### 3.3.2. Estimation of Serotonin Level

When compared with the disease control group, which had a substantial reduction in serotonin levels, animals in the levodopa plus carbidopa (standard)-treated group showed a highly significant (*P* < 0.001) improvement in serotonin levels (Figure 6(c)). The decrease in serotonin levels in Parkinson's disease is correlated with dyskinesia and mood disturbance. All dosage levels of PAME restored the diminished level of serotonin in a dose-dependent way with a substantially significant (*P* < 0.001) increase at the highest dose level, i.e., 800 mg/kg.

#### 3.3.3. Evaluation of Acetyl Cholinesterase (AChE) Activity

**Figure 7:**
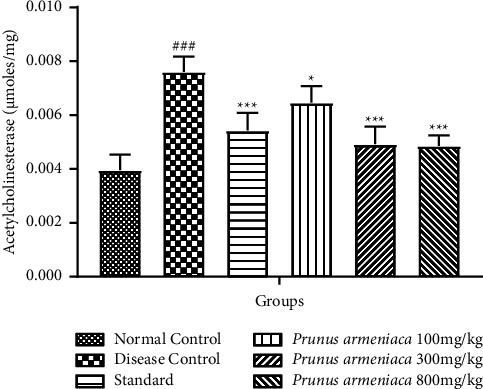
Effects of *P. armeniaca* L. on acetylcholinesterase levels in brain homogenates. Data are presented as mean ± S.E.M. (*n* = 6). ^$$$^*P* < 0.001 compared with the normal control group. ^*∗*^*P* < 0.05, ^*∗∗*^*P* < 0.01, and ^*∗∗∗*^*P* < 0.001 compared with the disease control group.

The AChE level was substantially increased (*P* < 0.001) in the disease control group. The standard treatment group showed a decrease in AChE levels (*P* < 0.001). A highly significant (*P* < 0.001) reduction in AChE levels was seen in the 800 mg/kg dose group of PAME, whereas, in the case of dose levels of 100 mg/kg and 300 mg/kg, a significant (*P* < 0.05) improvement was seen (Figure 7).

### 3.4. Evaluation of Oxidative Stress Parameters in the Brain

#### 3.4.1. Superoxide Dismutase (SOD) Levels

A significant reduction was found in the SOD levels of brain tissue homogenates after 21 days of experimentally induced parkinsonism with haloperidol (*P* < 0.001), but groups treated with PAME of 800 mg/kg had significantly enhanced the level of SOD after 21 days (*P* < 0.001) (Table 4). Even though the level of SOD was improved significantly with levodopa and carbidopa in haloperidol-treated rats, but this increment was not significant compared with extract-treated animals.

#### 3.4.2. Evaluation of Catalase (CAT) Level

As depicted in Table 4, when comparing the haloperidol-treated group with the normal control group for 21 days, there was a statistically significant (*P* < 0.001) reduction in catalase levels. The standard group significantly reversed the change in CAT levels caused by haloperidol. However, a dose-dependent significant improvement was observed in all the treatment groups such as 100 mg/kg (*P* < 0.001), 300 (*P* < 0.001), and 800 mg/kg dose levels (*P* < 0.001) when aqueous methanolic extract of PAME was administered for 21 days along with haloperidol administration.

#### 3.4.3. Evaluation of Reduced Glutathione (GSH) Level

Administration of haloperidol resulted in a significant depletion of GSH levels in brain tissue homogenates of the disease control group (*P* < 0.001), as shown in Table 4. The glutathione level reached near normal in the Parkinson's group who received levodopa plus carbidopa along with haloperidol. The recovery of GSH content in the treatment group with a dose of 800 mg/kg was highly significant (*P* < 0.001), while the other treatment doses (100 and 300 mg/kg) showed moderately significant improvement (*P* < 0.01), which was comparable to the replenishment of GSH level in the standard group (*P* < 0.05).

#### 3.4.4. Determination of Malondialdehyde (MDA) Levels

There was a significant (*P* < 0.001) increase in the level of MDA after exposure of rats to haloperidol in comparison with the normal control group. Concurrent treatment with aqueous methanolic extract of the plant had significantly reduced MDA levels (*P* < 0.05) at all the treatment doses. However, the standard treatment group exhibited a significant decrease after receiving treatment with levodopa plus carbidopa for 21 days, which was close to the normal control group (Table 5).

#### 3.4.5. Measurement of Nitrite Levels

PAME when injected at 100 mg/kg indicated a significant reduction (*P* < 0.05), whereas 300 and 800 mg/kg dose levels exhibited a significant decrease in nitrite levels (*P* < 0.001), as shown in Table 5. A significant reduction in the nitrite level was observed in the standard treatment group (*P* < 0.001). However, haloperidol was able to decrease the level of nitrite in the disease control group.

#### 3.4.6. Estimation of Total Protein Levels

When compared with the normal control group, tissue protein levels in the disease control group were significantly lower after treatment with haloperidol alone (*P* < 0.001). The groups of animals treated with different doses of plant extract showed recovery in the level of protein. The highest dose of PAME 800 mg/kg showed the statistically maximum improvement in the level of protein (*P* < 0.05). A more significant increase was observed in the protein level of the standard treatment group after treatment with haloperidol and concurrent administration of levodopa plus carbidopa (*P* < 0.001) (Table 5).

#### 3.4.7. Histopathological Examination of Brain Tissue

**Figure 8:**
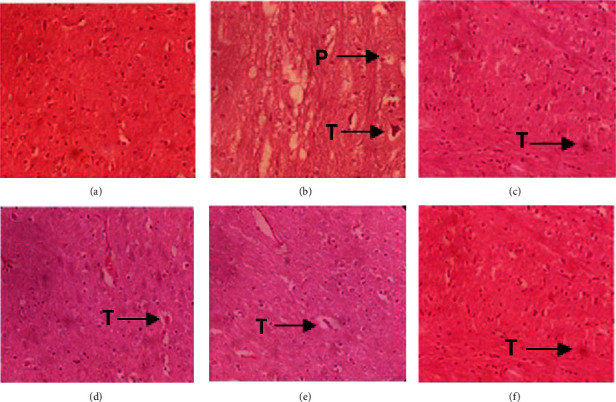
Histopathological examination of brain tissue: (a) normal control; (b) disease control; (c) standard; (d) extract 100 mg/kg; (e) extract 300 mg/kg; and (f) extract 800 mg/kg. The pictures were taken at 40×. T: neurofibrillary tangles; P: plaques.

The histopathological changes in brain specimens from various treated groups are displayed in Figure 8. Microscopic examination with a light microscope at 40× of brain tissues from the normal control group showed an intact histological structure and no histopathological alterations. Animals in the disease control group, on the other hand, showed neuronal degeneration, mild congestion in blood vessels, and slight hemorrhage. In the brain tissues, neurofibrillary tangles and plaques were also noticed in the haloperidol-treated group. Furthermore, an improvement in histological alterations was seen in groups treated with PAME at 100, 300, and 800 mg/kg dose levels. The standard treatment group was significantly replaced with health active neurons.

## 4. Discussion

Parkinson's disease is a complex, age-related nervous system disorder featured by reduction in the level of dopamine and the loss of nerve cells in the substantia nigra pars compacta [5]. Presently, PD is considered the second most prevalent neurodegenerative disorder [37]. Clinically, motor dysfunction and dementia have been widely attributed to PD. Neurodegeneration and neuronal dysfunction are considerably modulated by oxidative stress and neuroinflammation, leading to the reduction of various antioxidants. The reported prevalence of PD increases from 1 to 5% with an increase in age from 65 to 85 years, respectively, and is more occurring in men than women [5,38], and this gender discrimination is attributed to the neuroprotective effect of estrogen in females [39–41]. The neuropathological mechanisms of PD are multifactorial and include genetic and nongenetic as well as environmental factors, while the etiology of PD is still largely unknown. Protein aggregate accumulation, mitochondrial damage, impaired protein clearance pathways, neuroinflammation, oxidative stress, excitotoxicity, and genetic mutations are the main pathological mechanisms. The current medication of PD treats only symptoms, neither slows down nor halts the dopaminergic neurodegeneration [42]. Numerous efforts have been made to discover, identify, and formulate disease-modifying agents, but these efforts are still restricted to symptomatic treatment. Regarding the treatment of PD, many guidelines are available in which dopamine agonist is used in treating young-onset patients and levodopa for older patients. For initial therapy, patients who go through first motor fluctuations, MAO-B inhibitors act as a better treatment option. Similarly, COMT inhibitors enhance the action of levodopa if any wearing-off symptoms present [10]. There are many side effects of the currently available anti-Parkinson drugs, so the current approaches for treatment focuses on newer agents that will either inhibit or terminate the progression of the ailment and be economical. Therefore, the need of developing new drugs from plant origin has preventive yet lesser side effects against Parkinson's disease. Plants with potent antioxidant activities have been a long-established source of potential bioactivity moieties with neuropharmacological activities. Quercetin, a potent antioxidant flavonoid, is present in *Prunus armeniaca*. Quercetin, a flavonoid-derived plant flavanol, has recently been discovered to have neuroprotective properties against neurodegenerative diseases. It has been proposed as a Parkinson's disease supplemental therapy, and the role of flavonoids in PD treatment has been extensively researched. It has pharmacological effects in PD by regulating various molecular pathways [43]. It also has therapeutic potential for the prevention and treatment of neurodegenerative diseases such as Alzheimer's disease (AD) and Parkinson's disease (PD) due to its antioxidant and anti-inflammatory properties, as well as its ability to cross the blood-brain barrier [20].

This research was carried out to assess the neuroprotective potential of the methanolic extract of *Prunus armeniaca* (PAME), and the standard drug that was used throughout the study was L-dopa plus carbidopa to compare the neuroprotective effect of our methanolic extract with current strategies and medications being used conventionally. Catalepsy is a key biomarker for the evaluation of Parkinson's disease, and this behavior was found to be improved and attenuated upon treatment with PAME (Figure 1). The extract understudy improved the muscle strength of experimental rats in a dose-dependent manner. It was found that, in a hanging test, PAME improved neuromuscular strength, which was compromised by prolonged haloperidol administration. The motor coordination was also found to be improved by extract administration as it was shown to increase the horizontal bar test time (Figures 2 and 3). The hazardous effects of oxidative stress are inhibited by the natural antioxidants present in different plants owing to the presence of phenolic compounds, flavonoids. The current study suggested that physical strength, balance, and coordination were improved and maintained by administration of extract. In this study, the preliminary qualitative analysis has revealed large fractions of compounds such as polyphenols, flavonoids, and alkaloids in the PAME, which were also present abundantly in different plants in previous studies showing neuroprotective effects [44]. Considering the antioxidant potential of *P. armeniaca* L. plant, we conducted an antioxidant assay of the extract to confirm the presence of this compound in sufficient quantity to show antioxidant properties. The antioxidant capacity of the extract was determined using the DPPH assay, and it was confirmed that PAME has an excellent antioxidant potential (Table 5). Different behavior analyses such as open field, hole board, narrow beam, swim, elevated plus maze, and catalepsy tests were performed, and the changes in the behavior of experimental animals were evaluated (Figures 3–6). All behavior parameters of experimental animals were improved in response to administration of PAME and standard drugs. Administration of the extract resulted in improvement of locomotor activity, motor coordination, exploratory activities, and reduction in depression, anxiety, and reduced episodes of catalepsy (Figures 3–6). Numerous previous studies have shown that increased oxidative stress in the body and antioxidant enzyme imbalance can be related to development of neurodegenerative disorders [45]. Provoked oxidative stress and disturbed normal state of cells can lead to production of free radicals and peroxides, leaving toxic effects on brain cells. The increased level of free radicals and peroxides can lead to damaging of all cell components including lipids, proteins, and DNA. Haloperidol has been reported to deteriorate the levels of normal storage of antioxidant enzymes in the body by increasing oxidative stress on administration, which was indicated by increased lipid peroxidation and decreased SOD, CAT, and GSH that guard against oxidative stress [46]. One of the major causes of neurodegeneration is oxidative stress, and to evaluate the test doses, the levels of all oxidative stress biomarkers were measured. In the current study, PAME significantly restored the levels of antioxidant enzymes including SOD, CAT, and GSH (Table 4) in the body and reduced the elevated levels of nitrites and MDA (Table 5). As antioxidant enzymes were recovered within the body, it might be the reason of improvement in behavior and different brain functions. In the current study, different parameters were evaluated to estimate the neurotransmitter levels in the body and the results showed that the levels of neurotransmitters including dopamine, serotonin, and noradrenaline were significantly increased (*P* < 0.001), whereas the level of acetylcholinesterase was decreased significantly (*P* < 0.001) (Figures 6 and 7). Histopathological data showed improvement in histological alterations, such as neurofibrillary tangles and plaques (Figure 8). Data regarding the toxicity study performed on heart, kidney, and liver are given in supplementary data (Tables S1–S6). The incidence of side effects was inquired by biochemical analysis via LFTs and RFTs, and none of the abnormally raised values were determined (Tables S7 and S8). Nonsignificant differences in values among different groups were seen. Furthermore, the histopathological examinations also revealed normal histological findings for all vital organs such as heart, kidney, and liver (Figures S1–S3).

## 5. Conclusion

In this study, the therapeutic potential of PAME in Parkinson's disease was investigated. The antioxidant potential of PAME was evaluated through behavioral, biochemical, and histological analyses and estimation of neurotransmitter levels, and its anti-Parkinson's activity was confirmed. It has been found to improve motor function deficits, behavioral disturbances, and neurotransmitter levels in a haloperidol-induced PD rat model. It has been observed that, in this disease, oxidative stress can be reduced and antioxidant enzymes can be recovered. It was also discovered to inhibit the acetylcholinesterase enzyme's activity in the brain tissue. Therefore, it can be concluded that this PAME might have a potential in the treatment of PD, and further research in this regard may open a new era of research if the study can be extended to the molecular level.

## Figures and Tables

**Table 1:** Physicochemical analysis of *P. armeniaca* L.

Sr. no.	Physicochemical parameters	Percentage
1	Moisture content	7
2	Total ash content	22
3	Sulfated ash	52
4	Water-insoluble ash	39
5	Alcohol-insoluble ash	19
6	Water-soluble extractives	1.6
7	Alcohol-soluble extractives	5.8

**Table 2:** Phytochemical analysis of *P. armeniaca* L.

Phytochemical parameters	Quantity (mg/g)
Polyphenols	26.07 ± 0.71
Total alkaloids	43 ± 0.28
Total flavonoids	70.66 ± 0.45

**Table 3:** Effects of *P. armeniaca* L. on the forced swim test.

Groups	Dose (mg/kg)	Swimming (sec)	Climbing (sec)	Immobility (sec)
Normal control	—	146 ± 3.65	28.50 ± 3.17	65.50 ± 5.05

Disease control (Haloperidol)	1	98.50 ± 6.08^$$$^	15.50 ± 2.40^$^	121 ± 6.77^$$$^

Standard (Levodopa + Carbidopa)	100/25	145.50 ± 4.20^*∗∗∗*^	27.66 ± 2.87^*∗∗∗*^	79.83 ± 5.44^*∗∗∗*^

*Prunus armeniaca* L. (PAME)	100	125.16 ± 3.95^*∗*^	25.67 ± 2.96^*∗∗*^	109.16 ± 6.17^*∗*^
	300	138 ± 2.84^*∗∗*^	18.5 ± 3.65^*∗∗*^	93.5 ± 10.54^*∗∗*^
	800	142.83 ± 3.04^*∗∗∗*^	21.5 ± 3.45^*∗∗∗*^	84.16 ± 4.81^*∗∗∗*^

Data are presented as mean ± SEM (*n* = 6). ^$^*P* < 0.05, and ^$$$^*P* < 0.001 in comparison with the normal control group. ^*∗*^*P* < 0.05, ^*∗∗*^*P* < 0.01, and ^*∗∗∗*^*P* < 0.001 in comparison with the disease control group.

**Table 4:** Estimation of SOD, CAT, and GSH levels in brain homogenates.

Groups	Dose	SOD (*μ*g/mg of protein)	CAT (*μ*mol/min/mg of protein)	GSH (*μ*g/mg of protein)
Normal control	—	2.189 ± 0.02	30.18 ± 0.1	12.186 ± 0.1

Disease control	1 mg/kg	1.324 ± 0.01^$$$^	21.58 ± 0.1^$$$^	9.366 ± 0.1^$$$^

Standard (levodopa + carbidopa)	100/25 mg/kg	2.171 ± 0.01^*∗∗∗*^	28.64 ± 0.1^*∗∗∗*^	11.664 ± 0.1^*∗∗*^

*Prunus armeniaca* L. (PAME)	100 mg/kg	1.569 ± 0.01^*∗*^	23.01 ± 0.3^*∗*^	9.779 ± 0.7^*∗∗*^
	300 mg/kg	1.832 ± 0.01^*∗∗*^	25.71 ± 0.3^*∗∗*^	10.437 ± 0.5^*∗∗*^
	800 mg/kg	2.035 ± 0.01^*∗∗∗*^	27.09 ± 0.4^*∗∗∗*^	11.172 ± 0.5^*∗∗∗*^

Data are presented as mean ± SEM (*n* = 6). ^$$$^*P* < 0.001 in comparison with the normal control group. ^*∗*^*P* < 0.05, ^*∗∗*^*P* < 0.01, and ^*∗∗∗*^*P* < 0.001 in comparison with the disease control group.

**Table 5:** Estimation of MDA, nitrite, and protein levels in brain homogenates.

Groups	Dose	MDA (nmol/mg of protein)	Nitrite (*μ*g/mg of protein)	Protein (*μ*g/mg)
Normal control	—	728 ± 2.4	2.01 ± 0.2	310.8 ± 1.2

Disease control	1 mg/kg	865 ± 1.5^$$$^	3.89 ± 0.2^$$$^	214.8 ± 1.5^$$$^

Standard (levodopa + carbidopa)	100/25 mg/kg	741 ± 1.7^*∗∗∗*^	2.23 ± 0.2^*∗∗∗*^	295.9 ± 1.2^*∗∗∗*^

*Prunus armeniaca* L.	100 mg/kg	823 ± 1.5^*∗∗*^	3.42 ± 0.2^*∗∗*^	240.2 ± 1.6^*∗*^
	300 mg/kg	791 ± 1.2^*∗∗*^	2.97 ± 1.2^*∗∗∗*^	273.1 ± 1.6^*∗∗*^
	800 mg/kg	765 ± 1.2^*∗∗*^	2.48 ± 0.2^*∗∗∗*^	287.7 ± 1.6^*∗∗∗*^

Data are presented as mean ± SEM (*n* = 6). ^$$$^*P* < 0.001 in comparison with the normal control group. ^*∗*^*P* < 0.05, ^*∗∗*^*P* < 0.01, and ^*∗∗∗*^*P* < 0.001 in comparison with the disease control group.

## Data Availability

The data used to support the findings of this study are available from the corresponding author upon reasonable request.

## Supplementary Materials

Supplementary Materials
*Prunus armeniaca* L. methanolic extract (PAME) and toxicity studies performed on the heart, liver, and kidney. All the toxicity data are provided in the supplementary material file and cited in the relevant section of the main text and supplementary material file.
