# Supplementary material for: Pharmacological Potential of the Standardized Methanolic Extract of Prunus armeniaca L. in the Haloperidol-Induced Parkinsonism Rat Model
Source: Evid Based Complement Alternat Med. 2022 Sep 29;2022:3697522. doi: 10.1155/2022/3697522 (PMC9536922; doi:10.1155/2022/3697522)
Supplement: Supplementary Materials — Prunus armeniaca L. methanolic extract (PAME) and toxicity studies performed on the heart, liver, and kidney. All the toxicity data are provided in the supplementary material file and cited in the relevant section of the main text and supplementary material file. [file 3697522.f1.docx]

**Supplementary data**

### Pharmacological potential of Standardized Methanolic Extract of *Prunus armeniaca* L. in haloperidol induced parkinsonism rat model

### 1. Estimation of SOD, CAT, GSH, MDA, Nitrite and Proteins levels in Heart

### 1.1. Super Oxide Dismutase (SOD) Levels

Administration of haloperidol resulted in significant depletion of SOD levels in brain tissue homogenate of disease control group (*P* < 0.001) as displayed in Table S1. The SOD level reached near normal in Parkinson group who received levodopa + carbidopa along with disease inducing agent (haloperidol). The recovery of SOD content was extremely significant in the *P.* *armeniaca* L*.* 100, 300, and 800 mg/kg treatment groups, and was comparable to the replenishment of SOD level in the standard group (*P* < 0.05, *P* < 0.01, *P* < 0.001), respectively.

### 1.2. Catalase (CAT) Levels

When compared with normal control group, tissue catalase levels were significantly lower in the disease control group after treatment with haloperidol alone (P< 0.001). The groups of animals treated with different doses of plant extract showed recovery in the level of catalase. The highest dose level i-e 800 mg/kg of *P. armenicae* L.showed statistically maximum improvement in the level of catalase (P< 0.001). However, other doses such as 100 and 300 mg/kg indicated statistically significant recovery in catalase level (P< 0.01). More significant increase was observed in CAT level of standard treated group after treatment with haloperidol and concurrent administration of levodopa + carbidopa (P< 0.001) (Table S1).

### 1.3. Glutathione (GSH) Levels

A significant reduction was found in GSH level of brain tissue homogenate after 21 days of experimentally induced Parkinsonism with haloperidol (*P* < 0.001), but groups treated with *P. armeniaca significantly* raised the level of GSH when treated with 800 mg/kg dose level after 21 days (*P* < 0.001) (Table S1). Even though, the level of glutathione was improved significantly with levodopa and carbidopa in haloperidol treated rats, it did not significantly increase than the level of GSH in normal control group.

**Table S1. Estimation of SOD, CAT and GSH levels in Heart**

| **Groups** | **Dose** | **SOD**  **(µg/mg of protein)** | **CAT**  **(µmol/min/mg of protein)** | **GSH**  **(µg/mg of protein)** |
| --- | --- | --- | --- | --- |
| Normal Control | - | 1.890 ± 0.1 | 27.38 ± 0.2 | 22.36 ± 0.1 |
| Disease Control | 1 mg/kg | 0.124 ± 0.1^$$$^ | 23.58 ± 0.1^$$$^ | 19.372 ± 0.002^$$$^ |
| Standard | 100 mg/kg | 1.75 ± 0.1^***^ | 26.41 ± 0.2^***^ | 22.084 ± 0.2^***^ |
| *Prunus armeniaca* | 100 mg/kg | 0.471 ± 0.1^*^ | 24.21 ± 0.2^*^ | 19.719 ± 0.2^**^ |
|  | 300 mg/kg | 0.842 ± 0.1^**^ | 24.97 ± 0.2^**^ | 20.647 ± 0.3^**^ |
|  | 800 mg/kg | 1.368 ± 0.1^***^ | 25.59 ± 0.2^***^ | 21.652 ± 0.3^***^ |

Data represented as Mean ± SEM (n=6). ^$$$^*P* < 0.001 in comparison to normal control group^*^*P* < 0.05, ^**^*P* <0.01 and ^***^*P* < 0.001 in comparison to disease control group.

### 1.4. Malondialdehyde (MDA) Levels

*P. armeniaca* L., when injected at 100, 300 and 800 mg/kg dose levels, exhibited significant decrease in MDA levels (*P* < 0.05, *P* < 0.01, *P* < 0.001), respectively as shown in Table S2. A significant reduction in MDA level was observed in standard treatment group (P < 0.001). Whereas, haloperidol was able to decrease the level of MDA in disease control group.

### 1.5. Nitrite levels

There was significant (*P* < 0.001) raise in the level of nitrite after exposure of rats to haloperidol in comparison to normal control group. Concurrent treatment with aqueous methanolic extract of plant at 100 and 300 mg/kg doses had significantly reduced nitrite level (*P* < 0.001, *P* < 0.001) at all treatment doses. However, standard treatment group exhibited a significant decrease after receiving treatment with levodopa + carbidopa for 21 days which was close to the normal control group (Table S2).

### 1.6. Protein levels

As depicted by Table S2, when compared to the normal control group, haloperidol treatment resulted in a statistically significant (*P* < 0.001) reduction in protein levels after 21 days. The control group significantly reversed the haloperidol-induced change in protein levels. But a dose dependent improvement was observed in all plant extract treated groups with a maximum increase at 800 mg/kg dose level. Whereas, other treatment doses such as 100 and 300 mg/kg dose levels exhibited (*P* < 0.05, *P* < 0.01) increase in protein level, respectively, when aqueous methanol extract of *P. armeniaca* L. was administered for 21

**Table S2. Estimation of MDA, Nitrite and Protein levels in Heart**

| **Groups** | **Dose** | **MDA**  **(nmol/mg 0f protein)** | **Nitrite**  **(µg/mg of protein)** | **Protein**  **(µg/mg)** |
| --- | --- | --- | --- | --- |
| Normal Control | - | 384.3 ± 3.01 | 5.91 ± 0.07 | 644.8 ± 0.5 |
| Disease Control | 1 mg/kg | 496.4 ± 3^$$$^ | 9.73 ± 0.05^$$$^ | 586.8 ± 0.5^$$$^ |
| Standard | 100 mg/kg | 399.6 ± 3.1^***^ | 6.57 ± 0.02^***^ | 631.97 ± 0.2^***^ |
| *Prunus armeniaca* | 100 mg/kg | 461.8 ± 3.5^*^ | 9.45 ± 0.03^**^ | 597.26 ± 0.2^*^ |
|  | 300 mg/kg | 437.3 ± 3.7^**^ | 8.62 ± 0.02^***^ | 508.75 ± 0.3^**^ |
|  | 800 mg/kg | 413.7 ± 3.1^***^ | 7.37 ± 0.02^***^ | 523.54 ± 0.3 ^***^ |

Data represented as Mean ± SEM (n=6). ^$$$^*P* < 0.001 in comparison to normal control group^*^*P* < 0.05, ^**^*P* <0.01 and ^***^*P* < 0.001 in comparison to disease control group

## 2. Estimation of SOD, CAT, GSH, MDA, Nitrite and Proteins levels in Liver

### 2.1 Super Oxide Dismutase (SOD) Levels

A significant reduction was found in SOD level of brain tissue homogenate after 21 days of experimentally induced parkinsonism with haloperidol (*P* < 0.001), but groups treated with *P. armeniaca* L. significantly raised the level of SOD after 21 days (*P* < 0.001) at maximum dose level while, at 100 and 300 mg/kg dosage level significant improvement was seen (*P* < 0.05, (*P* < 0.01), respectively (Table S3). Even though, the level of SOD was improved significantly with levodopa and carbidopa in haloperidol treated rats, it did not significantly increase than the level of SOD in normal control group.

### 2.2 Catalase (CAT) Levels

As depicted by Table S3 when comparing the haloperidol treated group to the normal control group for 21 days, there was a statistically significant (P< 0.001) reduction in catalase levels. The control group significantly reversed the haloperidol-induced change in CAT level. But a dose dependent improvement was noted in all plant extract treated groups with moderately significant recovery (P < 0.01) at 100 and 300 mg/kg doses. A maximum increase (P< 0.001) at 800 mg/kg dose level when aqueous methanolic extract of *P. armeniaca* L. was administered for 21 days along with haloperidol administration.

### 2.3 Glutathione (GSH) Levels

Administration of haloperidol resulted in significant depletion of GSH levels in brain tissue homogenate of disease control group (*P* < 0.001) as shown in Table S3. The glutathione level reached near normal in Parkinson’s group who received levodopa + carbidopa along with disease inducing agent (haloperidol). GSH content recovery was highly significant in treatment groups receiving *P. armeniaca* L.at 300 and 800 mg/kg dosage levels, and was comparable to GSH replenishment in the control group (*P* < 0.01).

**Table S3. Estimation of SOD, CAT and GSH levels in Liver**

| **Groups** | **Dose** | **SOD**  **(µg/mg of protein)** | **CAT**  **(µmol/min/mg of protein)** | **GSH**  **(µg/mg of protein)** |
| --- | --- | --- | --- | --- |
| Normal Control | - | 5.89 ± 0.03 | 36.58 ± 0.1 | 17.36 ± 0.01 |
| Disease Control | 1 mg/kg | 3.32 ± 0.02^$$$^ | 29.98 ± 0.3^$$$^ | 14.37 ± 0.02^$$$^ |
| Standard | 100 mg/kg | 5.37 ± 0.03^***^ | 35.81 ± 0.2^***^ | 16.88 ± 0.02^***^ |
| *Prunus armeniaca* | 100 mg/kg | 3.91 ± 0.02^*^ | 32.01 ± 0.1^**^ | 14.81 ± 0.02^**^ |
|  | 300 mg/kg | 4.67 ± 0.04^**^ | 33.77 ± 0.2^**^ | 15.47 ± 0.03^***^ |
|  | 800 mg/kg | 5.08 ± 0.03^***^ | 34.97 ± 0.2^***^ | 16.55 ± 0.03^***^ |

Data represented as Mean ± SEM (n=6). ^$$$^*P* < 0.001 in comparison to normal control group^*^*P* < 0.05, ^**^*P* <0.01 and ^***^*P* < 0.001 in comparison to disease control group

### 2.4 Malondialdehyde (MDA) Levels

There was significant (P< 0.001) increase in the level of MDA after exposure of rats to haloperidol in comparison to normal control group. Concurrent treatment with aqueous methanolic extract of plant had significantly reduced MDA level (P< 0.001) at 300 and 800 mg/kg treatment doses. However, standard treatment group exhibited a significant decrease after receiving treatment with levodopa + carbidopa for 21 days which was close to the normal control group (Table S4).

### 2.5 Nitrite levels

*P. armeniaca* L*.*, when injected at 100 and 300 mg/kg dose levels, exhibited significant decrease in nitrite levels (*P* < 0.01) and statistically maximum reduction was noted (*P* < 0.001) as shown in Table S4. A significant reduction in nitrite level was observed in standard treatment group (*P* < 0.001). Whereas, haloperidol was able to decrease the level of nitrite in disease control group.

### 2.6 Protein levels

In brain tissues protein, when compared to the normal control group, levels in the disease control group were significantly lower after treatment with haloperidol alone (*P* < 0.001). The groups of animals treated with different doses of plant extract showed dose dependent recovery in the level of protein. The highest dose level of *P. armenicae* L. i.e., 800 mg/kg showed statistically maximum improvement in the level of protein (*P* < 0.001). Whereas, 100 and 300 mg/kg displayed significant increase in protein level (*P* < 0.05 and *P* < 0.01), respectively. More significant increase was observed in protein level of standard treated group after treatment with haloperidol and concurrent administration of levodopa + carbidopa (*P* < 0.001) (Table S4).

**Table S4. Estimation of MDA, Nitrite and Protein levels in Liver**

| **Groups** | **Dose** | **MDA**  **(nmol/mg of protein)** | **Nitrite**  **(µg/mg of protein)** | **Protein**  **(µg/mg)** |
| --- | --- | --- | --- | --- |
| Normal Control | - | 826.3 ± 3.2 | 11.69 ± 0.2 | 584 ± 4.5 |
| Disease Control | 1 mg/kg | 992.4 ± 3.6^$$$^ | 19.70 ± 0.1^$$$^ | 397 ± 4.5^$$$^ |
| Standard | 100 mg/kg | 854.68 ± 3.4^***^ | 17.09 ± 0.2^***^ | 539 ± 4.7^***^ |
| *Prunus armeniaca* | 100 mg/kg | 957.4 ± 2.6^**^ | 13.40 ± 0.1^**^ | 429 ± 3.2^*^ |
|  | 300 mg/kg | 911.8 ± 2.5^***^ | 15.27 ± 0.2^**^ | 477 ± 3.3^**^ |
|  | 800 mg/kg | 872.9 ± 2.5^***^ | 16.24 ± 0.3^***^ | 505 ± 3.3^***^ |

Data represented as Mean ± SEM (n=6). ^$$$^*P* < 0.001 in comparison to normal control group^*^*P* < 0.05, ^**^*P* <0.01 and ^***^*P* < 0.001 in comparison to disease control group

## 3. Estimation of SOD, CAT, GSH, MDA, Nitrite and Proteins levels in Kidney

### 3.1 Super Oxide Dismutase (SOD) Levels

Administration of haloperidol resulted in significant depletion of SOD levels in brain tissue homogenate of disease control group (*P* < 0.001) as displayed in Table S5. The glutathione level reached near normal in Parkinson’s group who received levodopa + carbidopa along with disease inducing agent (haloperidol). SOD content recovery was extremely significant in the *P. armeniaca* L. 800 mg/kg treatment group, and was comparable to SOD level replenishment in the control group (*P* < 0.001).

### 3.2 Catalase (CAT) Levels

When compared to the normal control group, catalase levels were significantly lower in the disease control group after treatment with haloperidol alone (P< 0.001). The groups of animals treated with different doses of plant extract showed recovery in the level of CAT. The highest dose level of *P. armenicae* L.showed statistically maximum improvement in the catalase level (*P* < 0.001). More significant increase was observed in CAT level of standard treated group after treatment with haloperidol and concurrent administration of levodopa + carbidopa (*P* < 0.001) (Table S5).

### 3.3 Glutathione (GSH) Levels

A significant reduction was found in GSH level of brain tissue homogenate after 21 days of experimentally induced Parkinsonism with haloperidol (P < 0.001), but groups treated with *P. armeniaca* Significantly increased the level of GSH after 21 days (P < 0.001) (Table S5). Even though the level of GSH was improved significantly with levodopa and carbidopa in haloperidol treated rats, it did not significantly rise than the level of SOD in normal control group.

**Table S5. Estimation of SOD, CAT and GSH levels in kidney**

| **Groups** | **Dose** | **SOD**  **(µg/mg of protein)** | **CAT**  **(µmol/min/mg of protein)** | **GSH**  **(µg/mg of protein)** |
| --- | --- | --- | --- | --- |
| Normal Control | - | 7.89 ± 0.01 | 22.38 ± 0.1 | 19.36 ± 0.2 |
| Disease Control | 1 mg/kg | 5.12 ± 0.01^$$$^ | 17.58 ± 0.1^$$$^ | 26.37 ± 0.2^$$$^ |
| Standard | 100 mg/kg | 7.25 ± 0.01^***^ | 21.61 ± 0.1^***^ | 25.48 ± 0.2^***^ |
| *Prunus armeniaca* | 100 mg/kg | 5.53 ± 0.001^**^ | 18.81 ± 0.1^*^ | 21.91 ± 0.2^**^ |
|  | 300 mg/kg | 6.37± 0.01^***^ | 19.47 ± 0.1^**^ | 22.76 ± 0.2^***^ |
|  | 800 mg/kg | 6.98 ± 0.01^***^ | 20.79 ± 0.1^***^ | 24.52 ± 0.1^***^ |

Data represented as Mean ± SEM (n=6). ^$$$^*P* < 0.001 in comparison to normal control group^*^*P* < 0.05, ^**^*P* <0.01 and ^***^*P* < 0.001 in comparison to disease control group

### 3.4 Malondialdehyde (MDA) Levels

*P. armeniaca* L., when injected at 100, 300 and 800 mg/kg dose levels, exhibited significant decrease in nitrite levels (P < 0.05, P < 0.01, P < 0.001), respectively, as shown in (Table S6). A significant reduction in nitrite level was observed in standard treatment group (P < 0.001). Whereas, haloperidol was able to decrease the level of nitrite in disease control group.

### 3.5 Nitrite levels

There was significant (P< 0.001) raise in the level of nitrite after exposure of rats to haloperidol in comparison to normal control group. Concurrent treatment with aqueous methanolic extract of plant had significantly reduced nitrite level (*P* < 0.001) at all treatment doses. However, standard treatment group exhibited a significant decrease (*P* < 0.001) after receiving treatment with levodopa + carbidopa which was close to the normal control group (Table S6).

### 3.6 Protein levels

As depicted by Table S6, when comparing the haloperidol treated group to the normal control group for 21 days, there was a statistically significant (P< 0.001) reduction in catalase levels. The standard group significantly reversed the change in protein level caused by haloperidol. But a dose dependent improvement was observed in all treated groups with a statistically significant increase (*P* < 0.01) at 100 and 300 mg/kg dose levels, whereas (*P* < 0.001) improvement was observed at highest dose level i-e 800 mg/kg, respectively, when aqueous methanolic extract of *P. armeniaca*  was administered for 21 days along with haloperidol administration.

**Table S6. Estimation of MDA, Nitrite and Protein levels in kidney**

| **Groups** | **Dose** | **MDA**  **(nmol/mg of protein)** | **Nitrite**  **(µg/mg of protein)** | **Protein**  **(µg/mg)** |
| --- | --- | --- | --- | --- |
| Normal Control | - | 654 ± 4.5 | 18.69 ± 0.2 | 484.3 ± 5.3 |
| Disease Control | 1 mg/kg | 497 ± 4.5^$$$^ | 23.97 ± 0.2^$$$^ | 236.4 ± 3.9^$$$^ |
| Standard | 100 mg/kg | 616 ± 5.0^***^ | 19.09 ± 0.3^***^ | 429.68 ± 5.3^***^ |
| *Prunus armeniaca* | 100 mg/kg | 529 ± 5.1^*^ | 23.17 ± 0.2^***^ | 271.8 ± 3.7^**^ |
|  | 300 mg/kg | 558 ± 5.0^**^ | 22.56 ± 0.3^***^ | 354 ± 3.7^**^ |
|  | 800 mg/kg | 594 ± 5.0^***^ | 21.14 ± 0.3^***^ | 388 ± 3.6^***^ |

Data represented as Mean ± SEM (n=6). ^$$$^*P* < 0.001 in comparison to normal control group^*^*P* < 0.05, ^**^*P* <0.01 and ^***^*P* < 0.001 in comparison to disease control group

.

### 4. Effects on Liver Function Tests

Liver function tests were performed and Serum bilirubin, Alanine Aminotransferase (ALT), Aspartate Aminotransferase (AST), and Alkaline Phosphatase levels were all investigated. Disease control group showed elevation in liver function enzymes due to treatment with haloperidol. In comparison with the normal control group, there was a significant difference in the levels of the disease control group. Standard group along with all treatment groups presented significant recovery in the levels of liver function tests which were closely related to normal control group (Table S7).

**Table S7. Liver Function Tests**

| **Groups** | **Dose** | **Serum Bilirubin**  **(mg/dL)** | **ALT**  **(U/L)** | **AST**  **(U/L)** | **Alkaline Phosphatase**  **(U/L)** |
| --- | --- | --- | --- | --- | --- |
| Normal Control | - | 0.23 ± 0.06 | 39.6 ± 0.91 | 51.5 ± 0.64 | 56.2 ± 0.53 |
| Disease Control  (Haloperidol) | 1 | 0.18 ± 0.06^$$$^ | 52.7 ± 0.81^$$$^ | 60.7 ± 0.85^$$$^ | 71 ± 0.9^$$$^ |
| Standard  (Levodopa+ carbidopa) | 100 | 0.23 ± 0.04^***^ | 43.9 ± 1.08^***^ | 54.9 ± 0.7^***^ | 67 ± 0.59^***^ |
| *Prunus armenica* | 100 | 0.20 ± 0.04^**^ | 46.3 ± 1.10^***^ | 58.5 ± 0.6^**^ | 56.9 ± 0.71^*^ |
|  | 300 | 0.21 ± 0.08^***^ | 44.8 ± 0.47^***^ | 56.2 ± 0.8^**^ | 63.5 ± 0.6^***^ |
|  | 800 | 0.22 ± 0.04^***^ | 41.4 ± 0.94^***^ | 55.6 ± 0.74^***^ | 65.4 ± 0.57^***^ |

Data represented as Mean ± SEM (n=6). ^$$$^*P* < 0.001 in comparison to normal control group^*^*P* < 0.05, ^**^*P* <0.01 and ^***^*P* < 0.001 in comparison to disease control group

### 5. Effects on Renal Function Tests

Renal function tests were performed and the levels of Blood urea, Serum creatinine and Serum uric acid were evaluated. When comparing the levels of determined parameters in the haloperidol-treated group to the normal control group, there were no significant differences. Standard group along with all treatment groups also presented non-significant difference when compared to disease control group (Table S8).

**Table S8. Renal Function Tests**

| **Groups** | **Dose** | **Blood Urea**  **(mg/dl)** | **Serum Creatinine**  **(mg/dl)** | **Serum Uric Acid**  **(mg/dl)** |
| --- | --- | --- | --- | --- |
| Normal Control | - | 0.63 ± 0.06 | 12.9 ± 0.19 | 6.3 ± 0.06 |
| Disease Control  (Haloperidol) | 1 | 0.66 ± 0.05 | 13.2 ± 0.23 | 5.9 ± 0.08 |
| Standard  (Levodopa+ carbidopa) | 100 | 0.63 ± 0.06 | 12.4 ± 0.18 | 6.2 ± 0.09 |
| *Prunus armeniaca* | 100 | 0.62 ± 0.06 | 11.7 ± 0.17 | 6.0 ± 0.08 |
|  | 300 | 0.63 ±0.06 | 12.0 ± 0.17 | 6.2 ± 0.06 |
|  | 800 | 0.63 ± 0.06 | 12.3 ± 0.16 | 6.2 ± 0.06 |

Data represented as Mean ± SEM (n=6). Ns= non-significant

### 6. Histopathological Examination of Liver Tissue

Figure S1 displayed histopathological examination of liver tissues of normal control, disease control, standard and treatment group. In normal control group liver tissues displayed intact hepatocytes, normal portal vein and sinusoids without any necrosis. While disease control group depicted hepatocyte vacuolization, inflamed portal vein and congested sinusoids. All treatment groups showed normalization of vacuolization and inflammation.

### 7. Histopathological Examination of Renal Tissue

Histopathological examination of renal tissues of normal control, disease control, standard and all treatment groups are shown in Figure S2. Normal renal tissue showed compact structure of glomeruli which was surrounded by bowman’s capsule and no cell infiltration was found. Whereas, disease control group exhibited cell infiltration and mild necrotic changes as well. All treatment groups displayed no cell infiltration and necrosis was also recovered.

### 8. Histopathological Examination of Heart Tissue

Histopathological findings of heart in normal control as well as all other groups under examination showed, normal branching with typical anastomoses in myocardium and centrally located vesicular nuclei. There was intact structure of cardiac muscles with no signs of dysregulation, inflammation, interstitial edema, necrotic changes, and fibrosis (Figure S3).

**
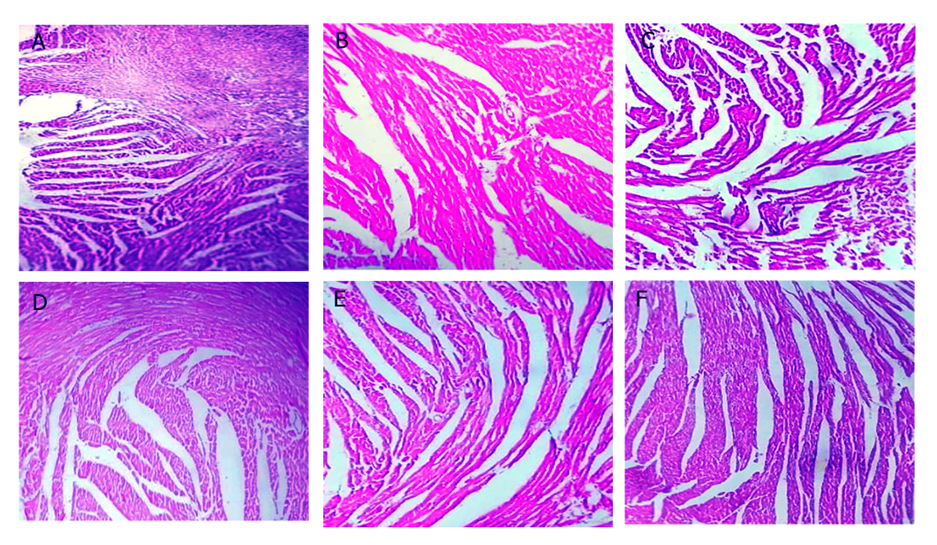
**

**Figure S1. Histopathological Examination of Heart Tissue The pictures were taken at 40 x.** (A) normal control; (B) disease control; (C) standard; (D) extract 100 mg/kg; (E) extract 300 mg/kg; (F) extract 800 mg/kg **CM**: Cardiac muscles

**
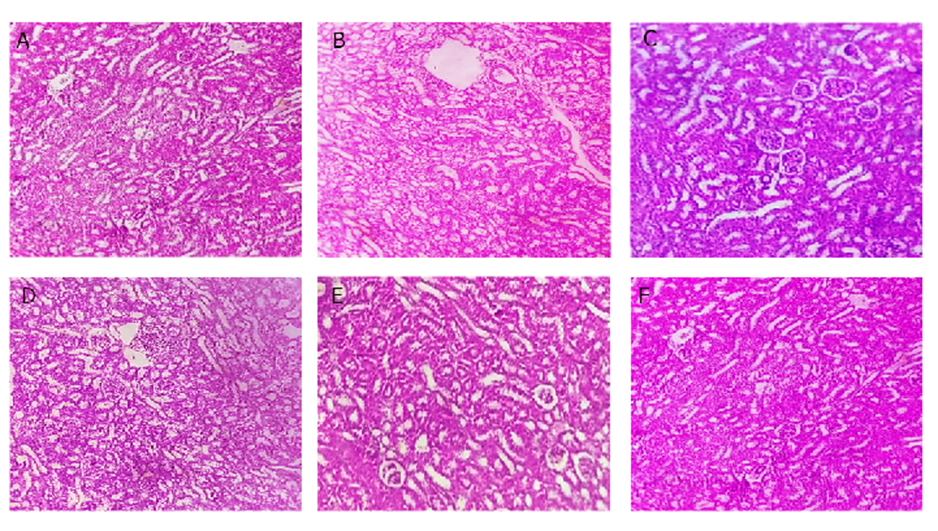
**

**Figure S2. Histopathological Examination of Kidney Tissue. The pictures were taken at 40 X.**(A) normal control; (B) disease control; (C) standard; (D) extract 100 mg/kg; **(**E) extract 300 mg/kg; (F) extract 800 mg/kg. **F:** Fibrosis, **G**: Glomeruli, **BC**: Bowman’s capsule


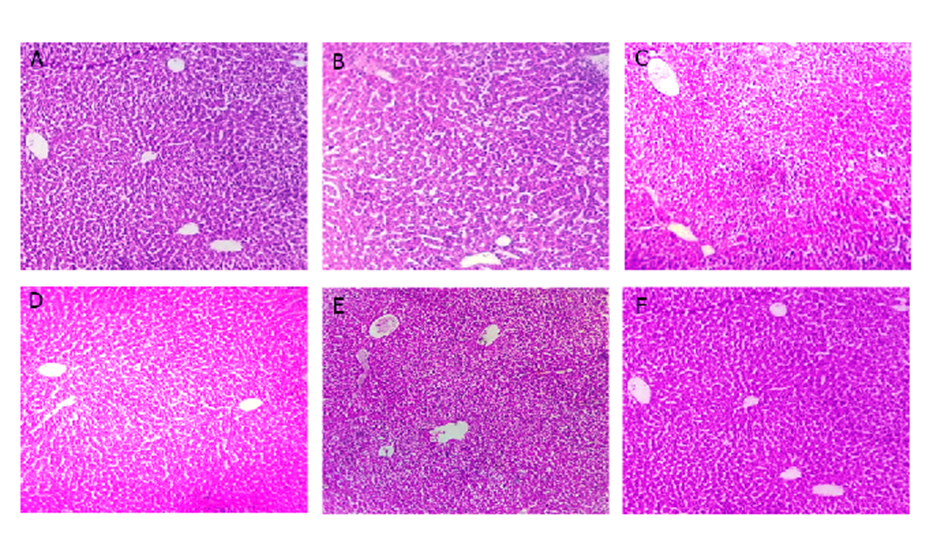


**Figure S3. Histopathological Examination of liver Tissue The pictures were taken at 40 x.**

(A) normal control; (B) disease control; (C) standard; (D) extract 100 mg/kg; (E) extract 300 mg/kg; (F) extract 800 mg/kg. **H**: Hepatocytes, **CV:** Central vein
